# Retraction: Team-based Learning and the Analytical Skills of Medical Students as a Consequence of Increased Problem Difficulty

**DOI:** 10.7759/cureus.r16

**Published:** 2019-08-06

**Authors:** Mustafa N Malik, Muhammad Abdullah Yousaf, Rida Riaz, Ahmed Ibrahim, Muhammad Abu Zar, Shehroz Aslam, Hafiz M Fazeel, Ceren Durer, Seren Durer

**Affiliations:** 1 Internal Medicine, District Headquarter Hospital, Rawalpindi, PAK; 2 Internal Medicine, Rawalpindi Medical University, Islamabad, PAK; 3 Internal Medicine, Nawaz Sharif Medical College - University of Gujrat, Gujrat, PAK; 4 Internal Medicine, Rawalpindi Medical University, Rawalpindi, PAK; 5 Hematology and Oncology, The University of Arizona, Tucson, USA; 6 Internal Medicine, Maricopa Medical Center, Phoenix, USA; 7 Internal Medicine, Florida Hospital, Orlando, USA

This article has been retracted at the request of the authors. A preliminary version of the article was previously published in an offline journal (and thus not detected by plagiarism software) and not properly credited by the authors nor disclosed to Cureus editorial staff.

